# Data in support on the shape of Schwann cells and sympathetic neurons onto microconically structured silicon surfaces

**DOI:** 10.1016/j.dib.2015.07.030

**Published:** 2015-07-31

**Authors:** C. Simitzi, P. Efstathopoulos, A. Kourgiantaki, A. Ranella, I. Charalampopoulos, C. Fotakis, Ι. Αthanassakis, E. Stratakis, A. Gravanis

**Affiliations:** a Foundation for Research and Technology-Hellas (F.O.R.T.H.), Institute of Electronic Structure and Laser (I.E.S.L.), Heraklion, Greece; b Department of Biology, University of Crete, Heraklion, Greece; c Department of Pharmacology, School of Medicine, University of Crete, Heraklion, Greece; d Department of Physics, University of Crete, Heraklion, Greece; e Department of Materials Science and Technology, University of Crete, Heraklion, Greece

## Abstract

This article contains data related to the research article entitled “Laser fabricated discontinuous anisotropic microconical substrates as a new model scaffold to control the directionality of neuronal network outgrowth” in the Biomaterials journal [1]. Scanning electron microscopy (SEM) analysis is performed to investigate whether Schwann cells and sympathetic neurons alter their morphology according to the underlying topography, comprising arrays of silicon microcones with anisotropic geometrical characteristics [1]. It is observed that although soma of sympathetic neurons always preserves its round shape, this is not the case for Schwann cells that become highly polarized in high roughness microconical substrates.

Specifications table

**Table:** 

Subject area	Bioengineering
More specific subject area	Microconical silicon substrates influence cell shape
Type of data	Figure
How data was acquired	Rat Schwann cells and superior cervical ganglia were cultured and SEM analysis performed
Data format	Analyzed
Experimental factors	3 substrates with increasing roughness were used
Experimental features	Shape of Schwann cells and soma for neurons was analyzed with SEM
Data source location	Heraklion, Greece
Data accessibility	Data is provided in the article

Value of data

• These data provide a more detailed study of the shape of the soma of sympathetic neurons compared to that of Schwann cells grown on microconically structured silicon surfaces of arbitrary and elliptical shape.
• Microconically structured silicon surfaces with anisotropic geometrical characteristics can be used to probe the directionality of neuronal cell cultures.
• Microconically structured silicon surfaces can be used to investigate the influence of surface topography on shape characteristics of other cell types.
• The SEM images provided here can serve as benchmark to other researchers who investigate the effect of culture surfaces with anisotropic geometrical characteristics on the shape of the soma of sympathetic neurons and Schwann cells.

## 1 Data, experimental design, materials and methods

Many studies report the response of different cell types which do not form extensive cell projections (i.e., fibroblasts, osteoblasts, Schwann cells, etc.) on artificial micro-and/or nanostructured surfaces [2–4]. In such cell types, the underlying topographical cue rearranges and reorganizes the cytoskeleton of the whole cell. In contrast to the above mentioned cell types, nerve cells respond to topographical cues in a quite distinct manner, mainly due to their unique morphology. The neuron consists of a network of long neurite projections (i.e., axons and dendrites), which extend from the cell body. When cultured on different substrates, the neurite projections have been shown to rearrange their network shape according to the imposed topography, while the cell body does not necessarily follow a similar adjustment [5].

We have shown that discontinuous anisotropic topographies such as this of microconically structured silicon surfaces influence cell growth orientation of Schwann cells and sympathetic neurons [1]. More specifically, neuronal axons and Schwann cells follow the directionality of the major axis of microcones with elliptical shape while there is a random orientation for substrates with microcones of arbitrary shape. In this article, we demonstrate via SEM microscopy on the different cell types the effects of the above mentioned topography on cell shape.

### 1.1 Cell culture on micropatterned Si substrates

#### 1.1.1 Dissociated Schwann cells

Schwann cell primary cultures were prepared from sciatic nerves of P2-P4 rat pups. Upon dissection, nerves were enzymatically triturated in a Trypsin 0.25% and 1% collagenase Type 1 (Sigma C0130) in HBSS solution for 30 min at 37 °C. The enzymes were deactivated by adding Dulbecco׳s modified Eagle׳s medium (DMEM; Invitrogen, Grand Island, NY, USA) containing 10% fetal bovine serum (FBS; Biosera, Sussex, UK) to the cells and the sciatic nerves were mechanically dissociated into single cell suspensions and plated to dishes. Before the first passage, Schwann cells were purified from contaminating fibroblasts using anti- thy1.1 antibody (AbD Serotec) and rabbit complement (Calbiochem) according to Brockes et al. [6]. Then 10^5^ cells were seeded onto micropatterned silicon scaffolds and cultured for 4–6 days until fixation for further analysis, with medium replacement every 2 days. In some experiments, PDL coating has been used.

#### 1.1.2 Dissociated superior cervical ganglia neuronal cultures

Superior cervical ganglia (SCG) were dissected from newborn (P0–P1) rat pups and dissociated in 0.25% trypsin for 30 min at 37 °C. After dissociation, SCG neurons were re-suspended in Roswell Park Memorial Institute (RPMI 1640, GIBCO/Life Technologies 32404-014) culture medium 1640 containing 10% FBS, 100 units/ml penicillin and 0.1 mg/ml streptomycin (p/s) (GIBCO/Life Technologies 15140-122), 10 mM antimitotic agent FdU with uridine, and 50 or 100 ng/ml Nerve growth factor (NGF; 2.5 S, Millipore, Billerica, MA, USA). 10^4^ cells were plated on collagen coated or micropatterned silicon scaffolds in 48-well plates and cultured for 7 days until fixation for further analysis. Medium was changed every 2 days.

#### 1.1.3 DRG explants

Mouse embryos (embryonic day (E) 13.5) were dissected from pregnant mice. DRGs from the lumbar region (L1–L4) were collected in cold PBS, then seeded onto the micro-structured Si substrates (2–3 DRG per substrate) and incubated in growth medium (DMEM)-F12, (GIBCO/Life Technologies 22320-022) supplemented with 10% FBS and 100 ng/ml NGF. Explants were incubated 5 days in vitro (DIV) until fixation for SEM analysis.

### 1.2 Scanning electron microscopy (SEM)

The morphology of the cells growing on the patterned surfaces was analyzed by SEM. After culture termination, the cells were washed with 0.1 M sodium cacodylate buffer (SCB) and then incubated in the same solution for 15 min. After repeating this step twice, the cells were fixed using 2% glutaraldehyde, 2% formaldehyde in 1% SCB fixative buffer for 1 h at 4 °C. All surfaces were then washed twice (for 15 min each time) with 1% SCB at 4 °C, dehydrated by immersion in serially graded ethanol solutions (50–100%) and incubated for 15 min in dry 100% ethanol. Prior to electron microscopy examination, the samples were sputter-coated with a 10 nm gold layer (Humme Technics Inc, Alexandria, Virginia USA). SEM analysis used a JEOL 7000 field emission scanning electron microscope at an acceleration voltage of 15 kV.

### 1.3 Results

**Fig. 1:**
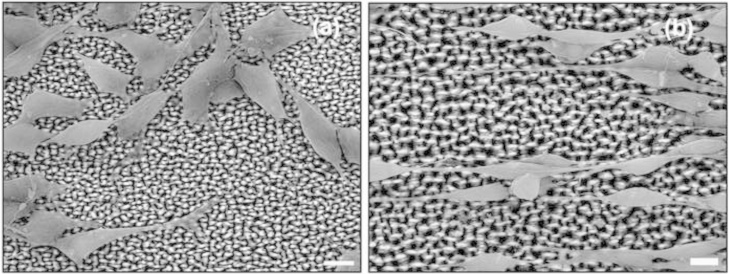
Top views of scanning electron microscopy images of Schwann cells on low (a) and medium (b) roughness micropatterned substrates. Scale bar: 10 μm.

Although arbitrary Schwann cell growth takes place on the microconically structured silicon surfaces of arbitrary shape (low laser energy, low roughness), Schwann cells tend to follow a preferential orientation when grown on substrates of microconically structured silicon surfaces of elliptical shape (medium and high laser energy, medium and high roughness) [1]. SEM study revealed a differential cell shape on the different substrates. Schwann cells grown on the surfaces comprising microcones (MCs) of arbitrary shape were mostly polygonal-shaped (Fig. 1a). On the contrary, the majority of the cells on the surfaces comprising MCs of elliptical shape were mostly spindle-shaped (Fig. 1b).

**Fig. 2:**
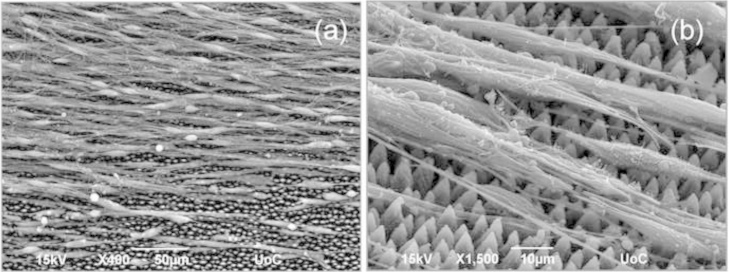
Spatial relationships between outgrown neurites and migrating Schwann cells emanating from a DRG explant positioned on medium roughness micropatterned Si substrates. (A) Top (a) and tilted (b) view of scanning electron microscopy images of Schwann cells and axons growing on top of them.

In the case of the whole DRG explants, when these were grown on the microconically structured silicon surfaces of elliptical shape (medium and high laser energy, medium and high roughness), SEM analysis revealed that the migrating Schwann cells were oriented parallel to the elliptic MCs (Fig. 2). Furthermore, most of the axons were grown on top of the aligned Schwann cells [1]. It is therefore postulated that the elliptical MCs of medium and high roughness substrates dictate a virtual path to Schwann cells, which in turn guide axonal outgrowth.

**Fig. 3:**
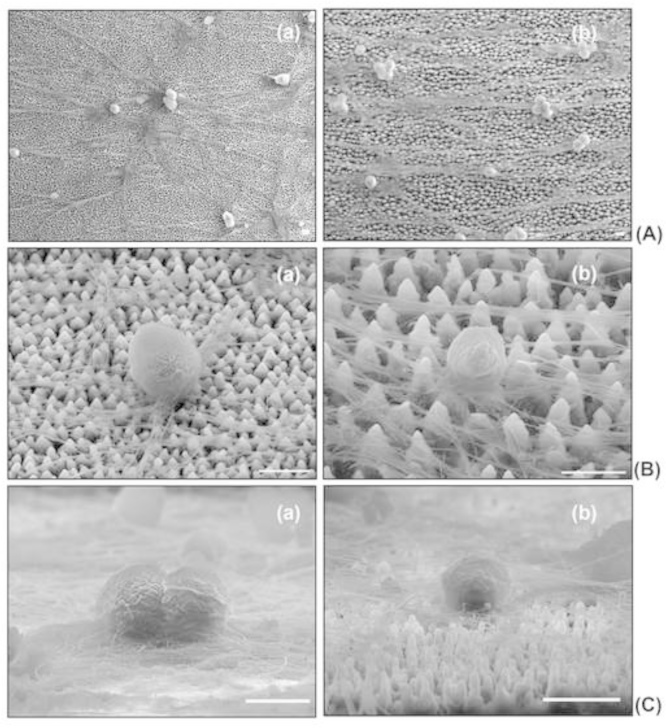
SEM of SCGs onto microconically structured Si surfaces substrates (A) Top views and (B)Tilted views of SCGs somas on low (a) and high roughness (b) micropatterned substrates. (C) Cross-sectional views of SCGs somas on flat (a) and medium roughness (b) Si surfaces. Scale bar: 10 μm.

In the case of sympathetic neurons, although the axons on the microconically structured silicon surfaces of arbitrary shape (low laser energy, low roughness) grew randomly, neurons on microconically structured silicon surfaces of elliptical shape (medium and high laser energy, medium and high roughness) exhibited a directional growth providing a network of parallelly aligned axons [1]. SEM microscopy revealed that the somas were always of round shape, regardless the type of the culture substrate used, i.e., flat or different microstructured surfaces (Fig. 3).
